# Subchronic Toxicological Study of Two Artemisinin Derivatives in Dogs

**DOI:** 10.1371/journal.pone.0094034

**Published:** 2014-04-16

**Authors:** Ji-ye Yin, He-mei Wang, Quan-jun Wang, Yan-sheng Dong, Gang Han, Yong-biao Guan, Ke-yong Zhao, Wen-sheng Qu, Ye Yuan, Xiao-xin Gao, Shu-fang Jing, Ri-gao Ding

**Affiliations:** Beijing Institute of Pharmacology and Toxicology, Beijing, China; Oswaldo Cruz Institute (IOC-Fiocruz), Brazil

## Abstract

The objective of our study was to profile and compare the systematic changes between orally administered artesunate and intramuscularly injected artemether at a low dose over a 3-month period (92 consecutive days) in dogs. Intramuscular administration of 6 mg kg^-1^ artemether induced a decreased red blood cell (RBC) count (anemia), concurrent extramedullary hematopoiesis in the spleen and inhibition of erythropoiesis in the bone marrow. We also observed a prolonged QT interval and neuropathic changes in the central nervous system, which demonstrated the cortex and motor neuron vulnerability, but no behavioral changes. Following treatment with artesunate, we observed a decreased heart rate, which was most likely due to cardiac conduction system damage, as well as a deceased RBC count, extramedullary hematopoiesis in the spleen and inhibition of erythropoiesis in the bone marrow. However, in contrast to treatment with artemether, neurotoxicity was not observed following treatment with artesunate. In addition, ultra-structural examination by transmission electron microscopy showed mitochondrial damage following treatment with artesunate. These findings demonstrated the spectrum of toxic changes that result upon treatment with artesunate and artemether and show that the prolonged administration of low doses of these derivatives result in diverse toxicity profiles.

## Introduction

Artemisinin, from sweet wormwood, and its derivatives have been shown to exert antimalarial activity and have consequently been developed as treatments for malaria caused by the *Plasmodium falciparum* parasite. They have also been shown to be effective against other parasitic infections, such as schistosomiasis. More recently, the potent anticancer properties of artemisinin compounds *in vitro* and *in vivo* have also been demonstrated [1]. Generally, artemisinin derivatives can be divided into two groups: ester derivatives, such as artesunate (ART), and ether derivatives, such as artemether (ARM) or arteether (ARE). While ART is water soluble, ARE and ARM are fat soluble; however, they are all rapidly converted to dihydroartemisinin (DHA) after administration [2].

Among the artemisinin derivatives, ART and ARM are the most commonly used. Recently, *P. falciparum* resistance to clinical treatment has increased; however, administration of ART and/or ARM still remains the most effective treatment for severe malaria [3], [4]. Research has also suggested that ART could be a potential treatment for *schistosoma* infection, *babesia* infection and *Toxoplasma gondii*, which leads to acute toxoplasmosis [5]–[9]. Furthermore, in mouse models, ART has been found to exert immunomodulatory effects in collagen-induced arthritis and attenuate the allergic response in asthma [10]–[12]. Promisingly, ARM has been shown to exert anticancer activity in tumor cell lines, while ART has shown anticancer properties in mice with established human tumors [13]–[18]. Several research groups have additionally reported that artemisinin derivatives have antiviral properties *in vitro* [19], [20]. However, clinical research regarding the toxic effects of these derivatives following a low dose treatment protocol over an extended time period is lacking. As such, it is the aim of this study to provide toxicological data for ART and ARM that may be used as a reference for future prospective clinical research studying the antischistosomal, general antiparasitic and anticancer activity of artemisinin derivatives, which would typically rely on a low dose treatment regime over an extended period of time. As ICH recommend that 3 months of repeated-dose toxicity studies in dogs can support marketing for 2 weeks to 1 month treatment duration, this 3 months’ duration in dog are considered long enough for those treatments[21].

While human subjects are safely administered doses of 2–8 mg kg^-1^ body weight per day for 3–5 days, most studies involving animals expose them to doses of (usually higher than) 25 mg kg^−1^ body weight for several consecutive days. The safety profiles of artemisinin drugs have previously been shown to be excellent [22]; however, concern regarding their potential neurotoxicity and hematotoxicity remains, especially in rodents, dogs and monkeys, following intramuscular injections of lipophilic ARM or orally administered ART at relatively high doses [23], [24].

Several publications have examined the toxicity profiles and dose-related effects of artemisinin derivatives, as shown in Table 1; however, these studies have typically only examined the side effects of these derivatives over comparatively short time courses, and a detailed examination of their effect over longer periods is lacking [16], [19], [25]. Despite the apparent advantages of preventing relapse and other promising clinical applications, no study has reported the effects of artemisinin and its derivatives on laboratory animals treated for a prolonged period.

**Table 1:** Published studies examining the toxicology of artesunate (ART) and artemether (ARM).

Species	Drug	Dose and application method	Effect	Reference
Dogs	ARM	20 mg kg^−1^ per day, i.m. for 30 days	Axonal damage in the cerebellar roof, pontine and vestibular nuclei, and in the raphe/ paralemniscal region; hypochromic, microcytic anemia	Classen et al., 1999 [30]
Rats	ARM	25 mg kg^−1^ per day, i.m. for 7 days	Damage of trapezoid nuclei, abnormalities in balance and coordination	Akinlolu and Shokunbi, 2008 [41]
Mouse	ARM/ART	300 mg kg^−1^ per day, p.o. for 28 days	Balance/gait disturbances and mortality	Nontprasert et al., 2000 [42]
Monkeys	ART	40 mg kg^−1^ per day, p.o. for 14 days	Reduced reticulocyte count	Clark et al., 2008 [43]

i.m.  =  intramuscular injection; p.o.  =  oral administration.

In the present study, we chose two typical artemisinin derivatives, ART and ARM, and investigated the *in vivo* systematic changes and lesions induced by the drugs when administered at a dose of 6 mg kg^−1^ for 13 weeks in dogs as a representative model. We also compared the toxicity of the two artemisinin derivatives when they were administered by two different routes (orally and intramuscularly), which allowed us to predict the clinical safety of these derivatives following consecutive usage.

## Materials and Methods

### Ethics statements

All animal work in this study was conducted and approved by the Institute of Animal Care and Use Committee of the National Beijing Center for Drug Safety and Evaluation Research. The protocol was approved by the Committee on the Ethics of Animal Experiments (Permit Number: 2011-38).

### Animals

Sixty-four (32 of each sex) young adult beagle dogs, aged approximately 24–32 weeks and weighing between 6.0 and 8.0 kg each, were purchased from Marshall BioResources (Certification No. SCXK(jing)2011-0003, ChangPing District of Beijing, China). The animals were housed singly in suspended stainless steel grid cages (60×60×60 inches) in an air-conditioned room with a set temperature of between 20°C and 25°C and a relative humidity of 40% to 70%. The ventilation provided a total air exchange rate of 10–15 times/h. The animals were maintained on 12-h light/dark cycles and were provided with commercial dog food and tap water *ad libitum*. All animals were permitted an acclimation period of approximately 2 weeks. Dogs that showed any clinical signs of disease, or clinical or pathological abnormalities during this period were not selected for the study.

### Drug administration

The first day of treatment was regarded as day one in the study. Prior to drug administration, the animals were randomly assigned by weight and sex to one of four treatment groups (8 dogs of each sex per group): an ART vehicle control group (0.9% physiological saline), ART-treated (6 mg kg^−1^ per day, orally [p.o.], administered once daily, Guilin No. 1 Pharmaceutical Factory, Guilin, People’s Republic of China), an ARM vehicle control group (peanut oil, intramuscular injection [i.m.]) and ARM-treated (6 mg kg^−1^ per day, i.m., suspended in peanut oil, Guilin South Pharmaceutical Co. Ltd., Guilin, People’s Republic of China). Oral administration was performed about 2 h after feeding. ARM was administered by a bolus intramuscular infusion through a 25-gauge needle at a constant volume of 2 ml kg^−1^ once a day into the gluteal and infraspinous muscles. Two dogs of each sex from each group were allowed to recover without further treatment for an additional 4 weeks. The weight of the dogs was assessed daily, and the amount of ART and ARM-administered was adjusted to ensure constant administration of the designated dosage.

### Clinical observations

All dogs were assessed once daily for mortality and toxicological or other clinical signs, including behavioral changes (such as tremor, gait impairment, balance disturbance and auditory impairment), appetite, excreta (urinalysis for specific gravity, ketone bodies and pH), balance and coordination. Standard and acceptable veterinary health care practices were used during this evaluation to ensure good health of the animals.

### Electrocardiography

Electrocardiograms were recorded using an Auto Electrocardiogram (ECG-9130P, Nihon Kohden Corporation, Japan). Recordings were taken from each dog, while conscious, every 2 weeks from week-1. Leads I, II, III, aVR, aVL and aVF were utilized. The electrocardiography (ECG) parameters included heart rate, PR, QRS and QT interval, and QRS and T wave amplitudes. As the PQ and QT intervals vary with heart rate, they were adjusted to an RR-interval of 500 ms, corresponding to a heart rate of 120 bpm using the following equation: QT_500_ = QT – 0.084×(RR – 500).

### Clinical pathology

Blood samples were collected from the femoral vein of each dog prior to treatment and every 2 weeks after the first day of treatment. Hematology parameters were measured using an automated hematology analyzer (Sysmex, XT-2000iv, Toa Medical Electronics Co. Ltd., Kobe, Japan) using standard methods. Test parameters included hemoglobin (Hb) levels, the hematocrit (Hct) fraction, erythrocyte (RBC) count, leukocyte (WBC) count, neutrophilic granulocyte fraction (NE%), monocyte fraction (MO%), lymphocyte levels (LY%), platelet count (PLT), reticulocyte count (RET%) and RBC morphology. The following clinical chemistry parameters were measured using a Hitachi-7180 Chemistry Analyzer (High-Technologies Corporation, Tokyo, Japan) and standard methods: glucose, blood urea nitrogen, inorganic phosphorus, creatinine, total protein, albumin, calcium, aspartate aminotransferase (AST), alanine aminotransferase, alkaline phosphatase, total bilirubin, creatine kinase (CK), sodium, potassium and chloride.

The differential granulocytic and erythrocytic series (erythroid, myeloid blast cell count and the red ratio) were determined manually from blood smears.

For toxicokinetic analysis, blood samples were collected on weeks 1, 2, 4, 8 and 13 at 0 (before dosing), 0.5, 1, 2, 4, 8, 12 and 24 h after dosing. Approximately 1 ml blood was drawn from the antebrachial, cephalic or jugular vein of three animals of each sex per time point and deposited into heparinized tubes for immediate centrifugation at 3000 rpm at 4°C for 10 min. The plasma was then immediately transferred to 2 ml polypropylene tubes and stored on dry ice. Samples were stored at −80°C until HPLC analysis [26].

### Pathologic examination

Termination of treatment was scheduled to occur on weeks 4, 8 (two interim necropsies) and 13, followed by a 4-week recovery period. For pathological examinations, the animals were euthanized within 24 h of the final drug administration with sodium pentobarbital, followed by exsanguination via the femoral artery. A gross necropsy was performed, including a thorough visual examination of all the internal organs. Weights were obtained for the brain, heart, liver, spleen, lung, kidneys, adrenals, thyroid/parathyroids, thymus, pancreas and ovaries/testes/prostate, and organ-to-body weight ratios were calculated. For each animal, representative samples from approximately 48 organs and tissues were taken and fixed in neutral buffered 10% formalin, except for the sternum, which was retained in Helly’s fixative (1000 ml distilled water, 25 g potassium dichromate, 10 g sodium sulfate, 50 g mercuric chloride, 50 ml 40% formaldehyde). Histopathological sections of all samples were prepared by embedding in paraffin wax, multiple-sectioning and staining with hematoxylin and eosin (HE). Samples of the left ventricular myocardium, right lateral hepatic lobe and right kidney from two animals of each sex from each group were fixed immediately after exsanguination in 2.5% glutaraldehyde, post-fixed in 1.0% osmium tetroxide, and embedded in plastic. Ultrathin liver, kidney and heart sections were examined with a Hitachi H-600 transmission electron microscope (TEM). All other tissues were examined by light microscopy.

After removal, the cardiac conduction system, including the sinoatrial node (SAN) and atrioventricular node (AVN), were selected by the Palate method [27]. The brain was sectioned at multiple transverse levels to obtain susceptible brain regions [24]. To improve the definition of the brain lesions, toluidine stains were applied on selected duplicate sections, and all preparations were examined under a light microscope. For pathologic examinations, degenerative neurons were evaluated on three randomly selected brain sections. The adjacent fields of neurons in each selected section were counted using a microscope at a magnification of 200×. Only cells exhibiting a distinct nucleolus were evaluated. The severity of the neuropathological lesions was defined as follows: the total number of neuropathological lesions per section area yielded a severity score from 0 to 4, where 0 = unaffected, 1 = minimally affected neurons with one degenerative cell per section, 2 = two degenerative cells per section, 3 = three degenerative cells per section and 4 = multiple degenerative areas per section.

### Statistical methods

As only two groups of animals were involved for each administration route, a Student’s *t*-test was implemented. The results of all pair-wise comparisons were reported at 0.05 and 0.01 significance levels. All endpoints were analyzed using two-tailed tests unless otherwise indicated. For each sex, one-way analysis of variance (ANOVA) tests were conducted on the body weight, ECG measurements and hematology, clinical chemistry, coagulation and organ weight data. If a significant *F* ratio was obtained (*p*≤0.05), Dunnett’s *t*-test was used for pair-wise comparisons with the control group.

## Results

### Animal observations

The results of ophthalmoscopic examinations of the study animals did not indicate any treatment-related effects. There were no observable toxicologically relevant clinical signs related to behavior during the experiment.

A summary of test article-related responses can be found in Table 2. In the ARM-injected group, the main clinical signs were skin thickening at the injection site and myonecrosis-like features after treatment for 4 weeks up to the end of the recovery period.

**Table 2:** Overview of treatment-related observations during the study in male and female dogs.

Week of study	Week 4		Week 8		Week 13	
Treatment group	ART	ARM	ART	ARM	ART	ARM
Animals number of each sex	8	8	6	6	4	4
Heart rate	↓↓	-	↓	-	↓	-
Prolonged QT intervals	-	↑↑	-	↑	-	↑
Clinical chemistry	↑TP(F), ↓Alb(F)	↑↑AST, ↑↑CK, ↓↓Alb	-	↑↑AST, ↓↓Alb	↓TP(F), ↓Alb(F)	↑↑AST(M)
Hematology	↓Hct, ↓Hb	↑WBC, ↑NE%, ↑↑MO%, ↓Hct(F)	↓Hct, ↓Hb, ↓MCV	↑NE%, ↑MO%(M), ↑RET%(F), ↓Hct(F), ↓LY%	↓Hct, ↓Hb	-

“-” = No effect; M = male; F = female; ↑↑or↓↓ = statistically significant increase or decrease compared with the control group (Student’s *t*-test, *p*<0.01);↑or ↓ = statistically significant increase or decrease compared to the control group (Student’s *t*-test, *p*<0.05).

**Figure 1:**
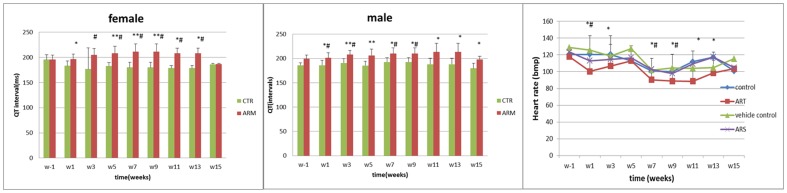
Effects of ARM and ART on QT intervals and heart rate. (A) Female dogs received 6 mg kg^−1^ ARM by intramuscular injection induced significant prolongation of QT intervals. (B) Male dogs received 6 mg kg^−1^ ARM by intramuscular injection induced significant prolongation of QT intervals. (C) Dogs orally received ART showed the significant decrease of heart rate. Data present mean±SD. *: Statistically significant compared to the control group, *p*<0.05; **: statistically significant compared to the control group, *p*<0.01. #: Statistically significant compared to w-1, *p*<0.05; ##: statistically significant compared to w-1, *p*<0.01.

ECG indicated a trend toward prolonged QT intervals in the ARM-treated dogs and a decreased heart rate in the ART-treated dogs (Figure 1A–C). Quantitative evaluation of the ECG results, recorded from week 3 until the final drug treatment, indicated that the mean QT intervals were prolonged (adjusted for heart rate, QT_500_) in male and female dogs treated with ARM compared with the vehicle control animals. Furthermore, there was a statistically significant decrease in heart rate during treatment, which was significantly different from the control group in weeks 3 and 5 in males, and in weeks 5–9 in females (*p*<0.01), and significantly decreased in the late stages of treatment in those treated with ART (*p*<0.05).

### Body and organ weights

There were no statistically significant effects on the absolute or relative weights of the brain, heart, liver, spleen, lungs, kidneys, adrenals, thyroid/parathyroids, pancreas, ovaries, testes or prostate in dogs treated with either ARM or ART compared with their relative controls (data not shown, Hadidian, Z., 1964 [28]); however, a mild decrease in thymus weight was noted in weeks 4 and 8 in one of the two male dogs that received ARM intramuscularly.

### Plasma drug levels

Plasma ARM concentrations were measured after daily intramuscular ARM injections at 6 mg kg^−1^ for 13 weeks. The mean concentration–time profile is shown in Table 3. The maximum concentrations after dosing were 90.63±24.99 µg l^−1^ on day 1, 219.10±41.30 µg l^−1^ in week 4, 46.20±13.15 µg l^−1^ in week 8 and 43.85±10.79 µg l^−1^ in week 13. Using a 40.92 ng ml^−1^ concentration as the lowest observed neurotoxic effect level, as reported by Li et al. (2006), the dosage of ARM supplied up to week 4 may be of toxicological relevance, as after this time, the plasma concentrations of the drug began to decrease.

**Table 3:** Drug administration, maximum exposure levels (*C*_max_) and maximum exposure times (*T*_max_) for ART- or ARM-treated dogs.

Treatment group	ART	ARM
Route	p.o.	i.m.
Dose	6 mg kg^−1^	6 mg kg^−1^
AUC_4w,_ (µg h L^−1^)	1272.16±538.52	739.00±209.23
*C*_max, w4_(ng mL^−1^)	-	219.10±41.30
*T*_max, w4_	-	0.50±0.03
*T*_1/2, w4_	-	3.00±1.16
AUC_8w_ (µg h L^−1^)	580.07±211.29	10.92±11.59
AUC_93D_ (µg h L^−1^)	728.62±440.01	71.32±56.64

AUC = area under curve of the mean concentration–time profiles; *T*_1/2_ = biological half-life; w4 and 4w = week four; w8 and 8w = week 8; 93D = approximately week 13.

### Serum chemistry

There were significant increases in serum AST and CK in the ARM-administered group in weeks 4 and 6 post-treatment compared to the control group (*p*<0.01). There was a significant decrease in serum albumin levels in weeks 4 and 10 in the ARM group and in weeks 10 and 12 in the ART group; however, the same changes could also be seen in the group treated with peanut oil only in weeks 4, 10 and 12. The changes in serum chemistry were found to be reversed at the end of the recovery period.

### Hematology

For the two treatment groups receiving ARM or ART, biological analysis showed that WBC count and NE% significantly increased, while LY% decreased compared to the control groups from week 2 until week 10 post-treatment (*p*<0.05); however, these parameters were also slightly changed in those animals receiving peanut oil alone compared to reference values from our lab.

In addition, there were significant increases in MO% in both males and females treated with ART after weeks 2 and 4 and in females treated with ARM after weeks 6, 8 and 10 (*p*<0.05; Table 2).

The RBC counts were found to decrease in week 2 (Table 2), with a corresponding decrease in Hb levels, among those treated with ART or ARM. In addition, Hct levels (in females only) decreased in week 2; however, this result was not significant compared to the control. There was a significant increase in RET% in week 10 in males and females that received ARM compared with the peanut oil control group (*p*<0.05). Other hematology parameters, including PLT and RBC morphology did not show significant changes over time (data not shown).

### Histopathology

Gross findings in the ART and ARM treatment groups were limited to the injection sites. Skin thickening at the injection site was observed in animals that received intramuscular injections. As expected, fascial inflammation, pseudocysts, muscle necrosis and hemorrhage were seen at the intramuscular injection sites, and histopathological data demonstrated that the drug exerted a cytotoxic effect on muscle cells close to the injection site. The severity of these lesions was graded by the a previously reported method [29], and the results showed that muscle tissue located at the site of the ARM injection was more severely inflamed than that in the vehicle control group (Table 4).

**Table 4:** Severity of muscle tissue injury at the injection site following repeated ARM or peanut oil administration by intramuscular injection in dogs (dose: 6 mg kg^−1^ for 13 weeks).

Week	Animal numbers (*n*)	ARM	Peanut oil control	Student’s *t*-test
W4	4	2.25±0.87	1.75±0.62	0.0409
W8	4	2.67±0.78	1.83±0.58	0.0086
W13	4	3.92±1.08	2.33±0.98	0.0070
W17	4	3.84±0.72	2.58±0.90	0.0015

Severity grades: 0 = no significant lesions; 1 = minimal; 2 = mild; 3 = moderate; 4 = marked; 5 = severe.

**Figure 2:**
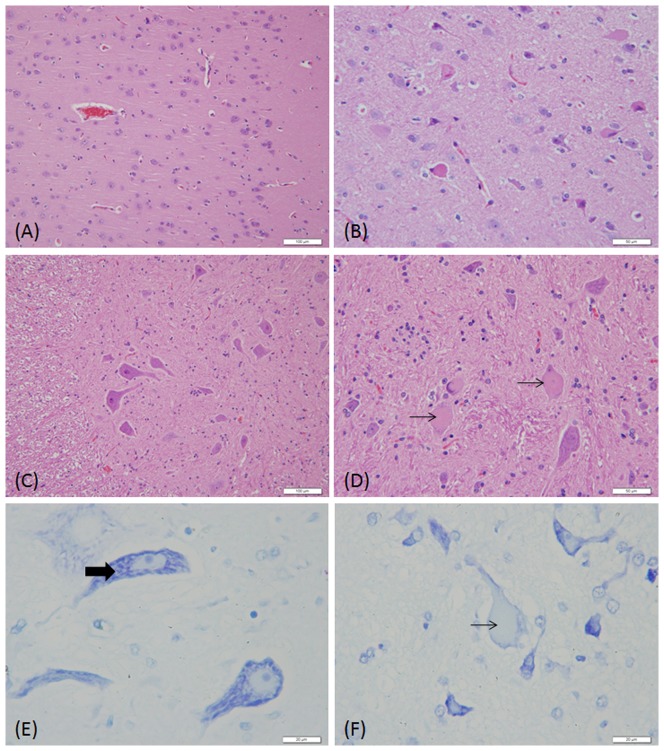
Comparison of the neuronal morphology in the cerebellar cortex and spinal cord of dog receiving vehicle only (A, C, and E) and ARM (6 mg kg^−1^ for 13 weeks) (B,D, and F). Hematoxylin and eosin staining show eccentric nuclear shrinkage and deep eosinophilia of the cytoplasm of nerve cells in the cerebellar cortex (black arrows, B), and nuclear shrinkage, karyolysis and eosinophilia of the cytoplasm in nerve cells in the spinal cord (black arrows, D). Toluidine blue staining show an absence of Nissl bodies in nerve cell in the pontine nuclei (black arrows, E), imaged with an oil immersion lens. (Original magnification of A and C×200, B and D×400, E and F×1000)

The brains of all dogs treated with ART and ARM were carefully examined. Neuropathological alterations were observed particularly in the group that received intramuscular ARM. Examples of neuronal damage compared to the vehicle controls are shown in Figure 2A–D. The observed abnormalities consisted of neuronal chromatolysis, swollen cell bodies, eccentric nuclei, neuronal degeneration and neuronal necrosis. These changes occurred 8 weeks after treatment with ARM. Neuropathological abnormalities were present in two of the four dogs (50%) examined in week 8, four of the four dogs (100%) examined in week 13, and one of the four dogs after the 4-week recovery period, when mild damage could still be seen (Table 5). Toluidine blue staining revealed an absence of Nissl bodies in the nerve cell bodies in the pontine nuclei (Figure 2E, F). The severity of damage to the trapezoid nucleus was expressed as both a numerical score and as the percentage of affected neurons in the cerebral cortex and spinal cord. However, the cortex and motor neuron showed a clear vulnerability. The mean severity score of the lesions in the brain for the ARM-treated group was 2.14; only a small number of degenerative cells were observed in this nucleus. No neuropathological abnormalities were detected in the other groups.

**Table 5:** Comparison of the positive pathological findings after ART or ARM administration.

Week of study	Week 4		Week 8		Week 13		Week 17	
Treatment group	ART	ARM	ART	ARM	ART	ARM	ART	ARM
Neuronal necrosis	0	0	0	2/4	0	4/4	0	1/4
Cardiac conduction system foci	0	0	1/4	0	2/4	0	0	0
Hepatocyte necrosis	0	3/4	0	2/4	0	0	0	0
Tubular cell degeneration	2/4	4/4	3/4	3/4	0	2/4	1/4	1/4
Extramedullary hematopoiesis	3/4	2/4	1/4	3/4	0	1/4	0	0
Inhibition of erythropoiesis	2/4	4/4	0	2/4	0	2/4	0	0

**Figure 3:**
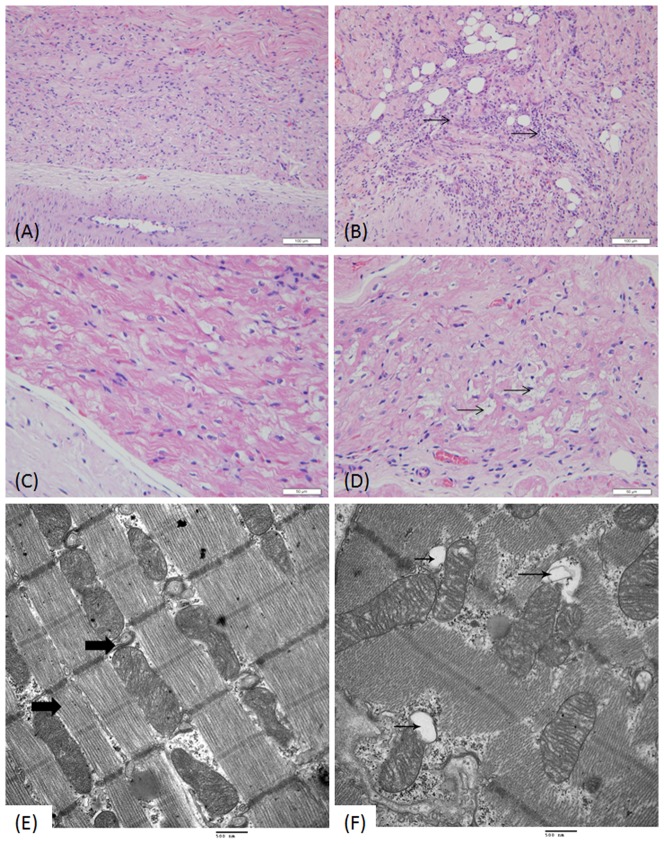
Comparison of the CCS morphology in the heart of dog receiving vehicle only (A, C, and E) and ART (6 mg kg^−1^) (B, D, and F). Hematoxylin and eosin staining show inflammation and fatty infiltration in the sinoatrial node (SAN) area of the cardiac conduction system (black arrows, B) and vacuolar degeneration of some pacemaker cells in the AVN (black arrows, D). Ultra-structural mitochondria in myocardial cells show mitochondrial bending, distortion, and swollen vacuoles (black arrows, F). (Original magnification of A and B×200, C and D×400, TEM image, 25,000×.)

When the SAN and AVN of the cardiac conduction system were examined, inflammation and fatty infiltrations into the SAN and major cell degeneration in the AVN were found following, in particular, ART treatment (Figure 3A–D).

**Figure 4:**
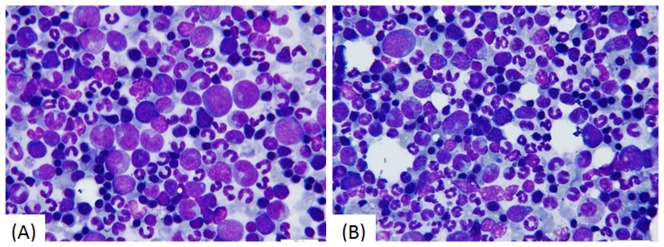
Hematopoietic cells in bone marrow smears (B) show a mildly increased proportion of granulocytes and a decrease of erythrocytes, while the percentage of middle–late stage erythroblasts (small round cells with dark blue nuclei) show a mild increase within the erythrocyte series. The sample was taken from a dog treated with 6^−1^ ARM i.m. for 13 weeks. (Stained with Wright’s stain and imaged with an oil immersion lens, 1000×.)

Histological examination of sternal bone marrow revealed mild inhibition of erythropoiesis and a decreased count of mature erythrocytes. Basophilic and polychromatophilic erythroblasts were the most frequently encountered erythroblast cell type (Figure 4A, B), and extramedullary hematopoiesis was observed in the spleen. These changes were observed in both the ART- and ARM-treated groups with no significant differences.

Light microscopy observations of liver sections revealed degenerative hepatocellular changes in some dogs treated with ARM, manifesting as cytoplasmic eosinophilia, vacuolation and condensed nuclei in some sporadic cells in the liver, although this was characterized as very mild. Histopathological changes in the kidneys included tubular epithelium vacuolation and necrosis in the ART- and ARM-treated groups compared to the control groups. These changes show that spontaneous lesions are common in the liver and kidney.

A variety of ultra-structural studies were carried out on the liver, kidney and heart following treatment with ART and ARM. Mitochondrial bending and distortion and swollen vacuoles could be seen in hepatocytes and myocardial cells as well as the proximal tubules in the kidney in weeks 4, 8 and 13 (Figure 3E, F).

## Discussion

The results of this study demonstrate the spectrum of toxic changes evoked by excessive exposure to artemisinin derivatives. Many of the toxic effects are specifically associated with ARM and include a decreased RBC count (*i.e*., anemia) and concurrent extramedullary hematopoiesis in the spleen, as well as the inhibition of erythropoiesis in the bone marrow. We also observed a prolonged QT interval and neuropathic changes in the central nervous system, which are indicative of cortex and motor neuron vulnerability; however behavioral changes were not observed. Following treatment with ART, we observed a decreased heart rate, which is most likely due to cardiac conduction system damage, a deceased RBC count, extramedullary hematopoiesis in the spleen and inhibition of erythropoiesis in the bone marrow. However, in contrast to treatment with ARM, neurotoxicity was not observed following treatment with ART. In addition, ultra-structural examination by TEM revealed mitochondrial damage. These findings demonstrate the spectrum of toxic changes that can appear upon exposure to artemisinin derivatives, such as ART and ARM. Interestingly, these toxic effects are observed in beagle dogs upon exposure to comparatively low doses and show that toxicity arises at doses far below 20 mg kg^−1^, which has previously been shown to induce toxicity [30].

A number of publications have revealed the clinical performance, toxicology and mechanism of action of artemisinin and its derivatives following short-term administration or administration in intervals. Xiao et al. (2002) revealed using hematology that rats treated orally with ARM at doses of 80 mg kg^−1^ and 400 mg kg^−1^ once every 2 weeks for 5 months showed a reversible reduction in the reticulocyte count and a reversible increase in Hb levels at both doses. No changes in the QT interval were reported. At a high dose of 400 mg kg^−1^, transient focal vesicle degeneration of the liver was observed, although there was no evidence of neuropathological alterations [31]. More recently, ART was administered to dogs for 7–385 days at a dose of 651–1178 mg m^−2^ (median, 922 mg m^−2^), and no neurological or cardiac toxicity was observed; however, a transient fever and hematological/gastrointestinal toxicity was noted in some of the 16 dogs involved in the trial [13].

Based on research regarding ARE (another derivative of artemisinin, which is similar to ARM), the calculated average minimal daily dose necessary to produce toxicity observable by histopathology in dogs is 6 mg kg^−1^ for 4 weeks [24], [32], [33]. Using this dosage, it was the aim of our study to investigate the time–effect relationship and compare the toxicity of ART and ARM-administered via different routes. In this regard, we found through long-term preclinical evaluation that ART and ARM treatment for 13 consecutive weeks induced minimal pathological changes.

However, the neurotoxic effects of these artemisinin derivatives and others have been a matter of concern for many years. Regardless of the mechanism of neurotoxicity, it is evident that it is highly dependent on the drug exposure time, rather than the exposure level (*C*_max_ and AUC) [29], and it can be concluded that the prolonged presence of ARM, due to its slow release from oil-based i.m. formulations, is the main cause of the toxicity observed in animals. The results of artemisinin neurotoxicity studies indicate that there are significant differences between the neurotoxic potential of these drugs depending on the route of administration. However, our study raises an interesting question: why did the level of ARM decrease in weeks 8 and 13? We supposed that injury to the blood vessels at the injection site contributes to a decrease in ARM absorption. In contrast, oral intake of ART or other artemisinin compounds, which is by far the most commonly used route, is followed by rapid clearance of these drugs and is thus likely to cause less toxicity in animals. This is in accordance with the results of Teja-Isavadharm et al. (1996) who studied the plasma concentrations of ART and its metabolite, DHA, and found that they declined much faster after oral administration than after an i.m. injection of ARM in an oil-based vehicle.

In a number of clinical trials studying the effects of artemisinin-type drugs on a large number of patients with malaria, adverse cardiovascular effects have not been noted. However, in animal models, such as beagle dogs and rats, where neurotoxicity associated with artemisinin-type drugs has been shown, prolonged QT intervals were noted to result from drug treatment [33]. In consideration of concurrent findings in the cardiac conduction system, it could be questioned whether the negative effects represent direct cardiotoxicity or are an indirect result of central nervous system toxicity, or both. In the present study and other animal research, it cannot be precluded that QT interval prolongation is caused by central nervous system toxicity [34].

The hematotoxicity of artemisinin derivatives has also been a subject of concern. The endoperoxide pharmacophore of artemisinins is responsible for the anti-proliferative effect on erythroid cells, and DHA or other derivatives are known to specifically target exponentially growing proerythroblasts and basophilic erythroblasts [35]–[37]. Consequently, artemisinins can inhibit the proliferation and differentiation of bone marrow hematopoietic stem and progenitor cells by inducing apoptosis at low doses.

Both ART and ARM are converted to DHA after administration *in vivo* but the hydro- and lipophilic properties of ART and ARM, respectively, decide their routes of administration. Obviously, ART can be taken orally, which is convenient; in contrast, oil injections may act as a depot and result in sustained release of the drug from the site of injection, and this may induce more toxic effects [38]. In consideration of this, the lipophilic properties of ARM and its pharmacokinetic profile, which result in prolonged interaction with the central nervous system, likely form the basis of the drug’s neurotoxicity.

The *C*_max_ and AUC values have previously been noted to be markedly reduced due to drug-induced metabolism [39]. Burk et al. (2012) explored the capacity of antimalarial drugs to induce drug metabolism via activation of constitutive androstane receptors through ligand binding, providing an explanation for the lower toxic profile of some artemisinins, such as ART, which may induce cytochrome P450 genes in vivo. Cell membranes, mitochondria, endoplasmic reticulum appear to be most sensitive to carbon-centered free radicals produced by artemisinins [40].

In conclusion, our findings suggest that the prolonged administration of low doses of artemisinins produces similar, but specific, toxicological effects to comparatively high doses of the same drugs administered over short periods. The pharmacokinetic properties of the drugs determine their toxicological potential, and the exposure time plays a significant role in the severity of the side effects experienced following drug treatment, even at low levels. Questions regarding the safety of long-term drug use and dosing regimens should be answered by appropriate clinical studies focused on toxicity and drug indications in the near future.
